# Optimizing the Required Cathodic Protection Current for Pre-Buried Pipelines Using Electrochemical Acceleration Methods

**DOI:** 10.3390/ma14030579

**Published:** 2021-01-26

**Authors:** Nguyen-Thuy Chung, Min-Sung Hong, Jung-Gu Kim

**Affiliations:** School of Advanced Materials Engineering, Sungkyunkwan University, 300 Chunchun-Dong, Jangan-Gu, Suwon 440-746, Korea; chung.ngthuy@gmail.com (N.-T.C.); smith803@skku.edu (M.-S.H.)

**Keywords:** cathodic protection, buried pipelines, rust layer, electrochemical acceleration test, applied current density, required current

## Abstract

Several corrosion mitigation methods are generally applied to pipelines exposed to corrosive environments. However, in the case of pre-buried pipelines, the only option for corrosion inhibition is cathodic protection (CP). To apply CP, the required current should be defined even though the pipeline is covered with various oxide layers. In this study, an electrochemical acceleration test was used to investigate the synthetic soil corrosion of a pre-buried pipeline. Potentiodynamic polarization experiments were first conducted to ascertain the corrosion current density in the environment, and galvanostatic measurements were performed to accelerate corrosion according to the operating time. In addition, corrosion current density and the properties of the rust layer were investigated via potentiodynamic polarization tests and electrochemical impedance spectroscopy (EIS) tests. The variation in surface corrosion was subsequently analyzed via optical microscopy (OM) and X-ray diffraction (XRD) measurements. Finally, an empirical equation for the optimized CP current requirement, according to the pipeline service time, was derived. This equation can be applied to any corroded pipeline.

## 1. Introduction

Buried steel pipelines commonly play an important role worldwide as a means of transporting gases and liquids over long distances to meet several demands [1,2]. These pipelines are utilized in various soil environments, where they are exposed to chloride ions, humidity, and microbiological factors throughout their life, which could result in corrosion [2]. Corrosion is the main degradation mechanism that can reduce the structural integrity of buried pipelines [3]. The most basic method to protect a pipeline from corrosion is the application of an external coating to block corrosive ions, oxygen, and water [4]. However, the process of applying a coating on a pipeline should be evaluated at the initial stage, since it is nearly impossible to coat a pipeline that has already been buried. Therefore, additional methods should be considered to protect pre-buried pipelines.

Cathodic protection (CP) has been used in conjunction with organic coatings as the first method to control metal corrosion. In addition, CP can be utilized for pipelines that have already been buried [5]. In order for this approach to function adequately, it is important to calculate the required CP current according to the corrosion rate, operating time, environment, and other factors [6,7]. While it is quite simple to calculate the required current according to international standards when CP is applied at the initial stage, more thorough considerations are needed to implement the CP approach on corroded pipelines, since the rust layer could affect the surface properties of the structure. However, studies regarding the proper CP current required in pre-buried pipelines are still lacking [8]. The NACE Standard SP0408-2019, i.e., Cathodic Protection of Reinforcing Steel in Buried or Submerged Concrete Structures, indicates how the CP method should be used for buried and submerged pipelines [9]. However, the standard does not specify the CP current required for corroded pipelines according to the corrosion time. For this reason, the optimized CP current required for a buried pipeline is derived in this work according to the corrosion time in a soil environment.

In this study, electrochemical tests were conducted to calculate the optimized CP current required according to the corrosion time. Galvanostatic tests were also carried out to accelerate the corrosion for 0.25, 1.2, 2.5, 5, and 7.4 years; electrochemical impedance spectroscopy (EIS) and potentiodynamic polarization experiments were performed after each test. After the electrochemical investigations, the surface of the corroded specimens was analyzed via optical microscopy (OM) and X-ray diffraction (XRD). Finally, an equation for the optimized CP current requirement concerning the pipeline service time was derived from the test results.

## 2. Materials and Methods

### 2.1. Specimen and Solution Preparation

Carbon steel (SPW400) with 8.7 mm thickness, a general material utilized for buried pipelines, was employed as a test specimen; the specific composition is listed in Table 1 (Korean Standards). Before conducting the experiments, the surfaces of the specimens were polished with 600-grit silicon carbide (SiC) paper, rinsed in ethanol via ultrasonication, and finally dried with air. The corrosion environment consisted of a synthetic soil solution with pH value is 6.8 which the pH was conditioned by diluted HNO_3_ and NaOH., the chemical composition of which is listed in Table 2.

### 2.2. Electrochemical Tests

All electrochemical tests were based on a three-electrode system configuration. (VSP 300, Bio-Logic SAS, Seyssinet-Pariset, France). The test specimen consisted of a working electrode; a graphite rod and saturated calomel electrode (SCE) were used as the counter and reference electrodes, respectively. The exposed area of the specimen was 0.25 cm^2^. An open-circuit potential (OCP) was established within 30 min before the electrochemical tests. The corrosion properties of the steel pipeline were evaluated via potentiodynamic polarization tests performed from an initial potential of −0.4 VOCP to a final potential of 1.2 VSCE with a sweep rate of 1 mV/s. Based on the potentiodynamic polarization results, the acceleration time for galvanostatic testing was calculated according to Faraday’s law. EIS experiments were subsequently performed with a 20 mV amplitude in the frequency range from 100 kHz to 10 mHz, and potentiodynamic polarization tests were conducted to obtain the corrosion current density after the corrosion acceleration.

### 2.3. Surface Analysis

Optical microscopy (SZ61, Olympus, Tokyo, Japan) was used to investigate the relationship between thickness loss and corrosion time to determine the corrosion rate through thickness loss. The corrosion products were analyzed via XRD (D8 Advance, Bruker Co., Karlsruhe, Germany) The XRD patterns were obtained using a Cu Kα target at 50 kV and 250 mA over a 2θ range of 20–70° with a scan rate of 2° min^−1^.

## 3. Results and Discussion

### 3.1. Potentiodynamic Polarization and Galvanostatic Tests

**Figure 1:**
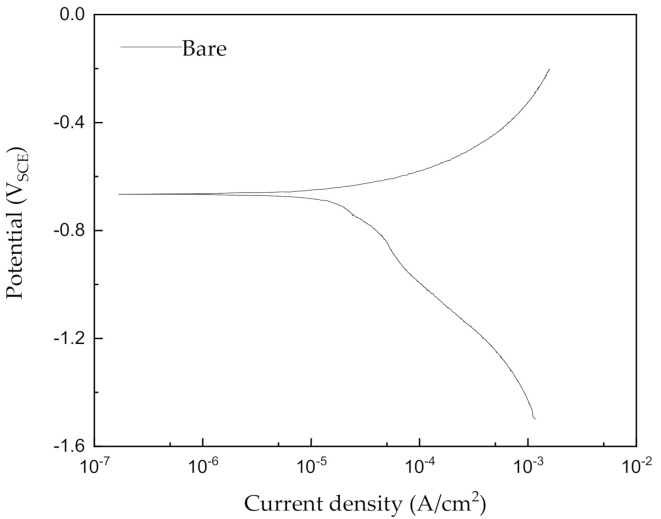
Potentiodynamic polarization curves for pipeline steel in a synthetic soil solution at room temperature.

To simulate testing over a period of years, the acceleration time should be calculated according to Faraday’s law [10]:

*Q* = *It* (1)

where *Q* is the total electric charge passed through the substance, *t* is the total time over which a constant current is applied, and *I* is the constant current for corrosion, i.e., the corrosion current density. According to Equation (1), as the total amount of electricity transferred through the specimen is kept constant, the time is reduced but the current must increase proportionally [11]. Therefore, in order to determine the experimental time, it is necessary to know the corrosion current density for a material in a given environment. The corrosion current density can be measured via potentiodynamic polarization testing. Figure 1 shows the potentiodynamic polarization curve of carbon steel in a synthetic soil solution, whereas the results of the potentiodynamic polarization test are displayed in Table 3. According to the test results, the specimen exhibits the corrosion current density of 4.2 $\mu A/{\mathrm{cm}}^{2}$, which leads to a corrosion rate of 0.098 mm/year. From this value, the acceleration time can be calculated using Equation (1). The values for the acceleration of the corrosion time depend on the purpose of the study, the applied current density, i.e., the acceleration current from the system, was set to 5000 $\mu A/{\mathrm{cm}}^{2}$, which is about 1,200 times larger than the corrosion current density. Corrosion times were set to be 0.25, 1.2, 2.5, 5, and 7.4 years in this study; all calculated values are listed in Table 4. These findings were applied to the galvanostatic tests in order to accelerate the corrosion of steel.

### 3.2. Surface Analysis

**Figure 2:**
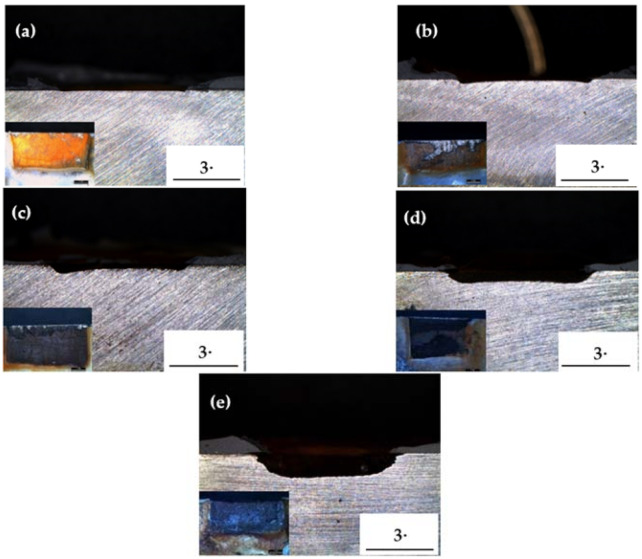
Cross-section images with magnification are x10 and x45 of the specimens after acceleration tests of (**a**) 0.25 years, (**b**) 1.2 years, (**c**) 2.5 years, (**d**) 5 years, and (**e**) 7.4 years.

**Figure 3:**
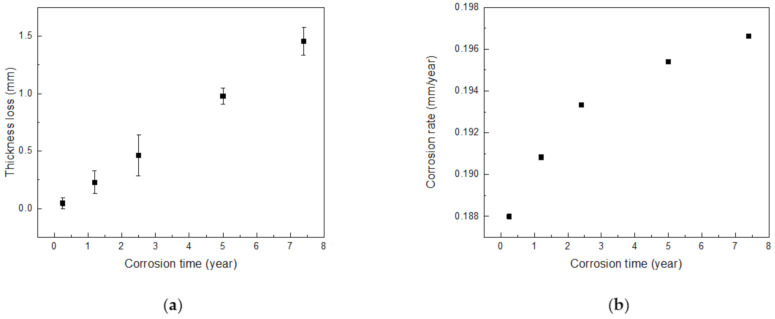
(**a**) Thickness loss of pipeline steel as a function of the corrosion time and (**b**) instantaneous corrosion rate of carbon steel as a function of the corrosion time.

After performing the acceleration tests using galvanostatic methods, surface analysis investigations were performed via OM and XRD to measure the thickness loss and rust chemical composition. Through examining the cross-section of the specimens, the thickness loss during the corrosion process can be determined. The cross-sections of the accelerated specimens are shown in Figure 2. It can be seen that, as the corrosion time increases, more rust accumulates, and the thickness loss gradually becomes greater. As shown in Figure 3a, the thickness loss for pipeline steel as a function of the corrosion time continuously increases. The thickness loss increases with the corrosion time regardless of whether the corrosion rate is fast or slow. Therefore, in order to describe the corrosion rate, the thickness loss should be divided by the total corrosion time [8], as shown in Figure 3b. From this figure, it can be seen that the corrosion rate is accelerated according to the corrosion time. However, the corrosion rate does not increase linearly, since its slope decreases as the corrosion time increases. It can be predicted that it will remain stable after a certain corrosion time, as an effect of the corrosive product on the corrosion process. This result will be more clearly demonstrated in the following section.

**Figure 4:**
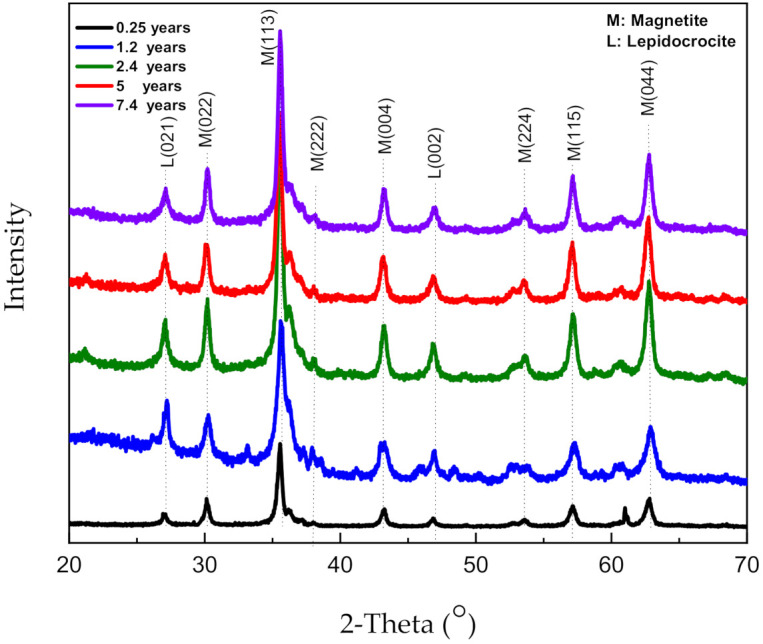
XRD patterns obtained for powdered rust formed after the galvanostatic tests.

To explore the influence of the corrosion products on the corrosion process, it is necessary to first determine the components of the rust layer. XRD measurements of the corrosion products were performed after completing the experiments. The corrosion rate at different exposure periods is related to the composition and structure of the rust layer. Figure 4 shows the XRD patterns of powdered rust on pipeline steel. Upon comparison to the reference patterns [12,13], it can be seen that the rust layers that form for different corrosion time durations exhibit the constituents that are mainly assigned to lepidocrocite (γ−*FeOOH*) and magnetite (Fe_3_O_4_) phases. In particular, the amount of lepidocrocite (brown rust) observed for the 0.25 and 1.2 years experiments accounts for a larger percent, it can visible to the naked eye. However, as the corrosion time increases, the amount of magnetite gradually increases. By contrast, Fe_3_O_4_ becomes the main component of the rust layer upon increasing the immersion time. The rapid Fe_3_O_4_ generation indicates that this material is directly converted from other corrosion products rather than being generated in the oxygen-depleted environment. Additionally, other studies have reported that Goethite (α−*FeOOH*) is located in the inner rust layer at the initial stage [14,15].

### 3.3. Electrochemical Acceleration Test Results

**Figure 5:**
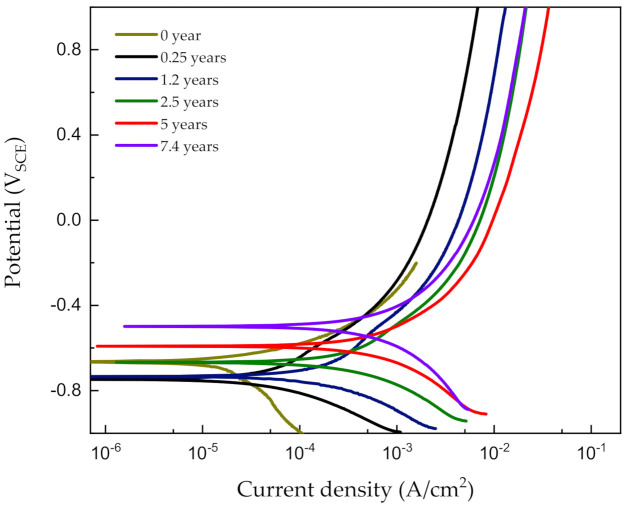
Potentiodynamic polarization curves of pipeline steel in a synthetic soil solution as a function of the corrosion time after galvanostatic tests.

**Figure 6:**
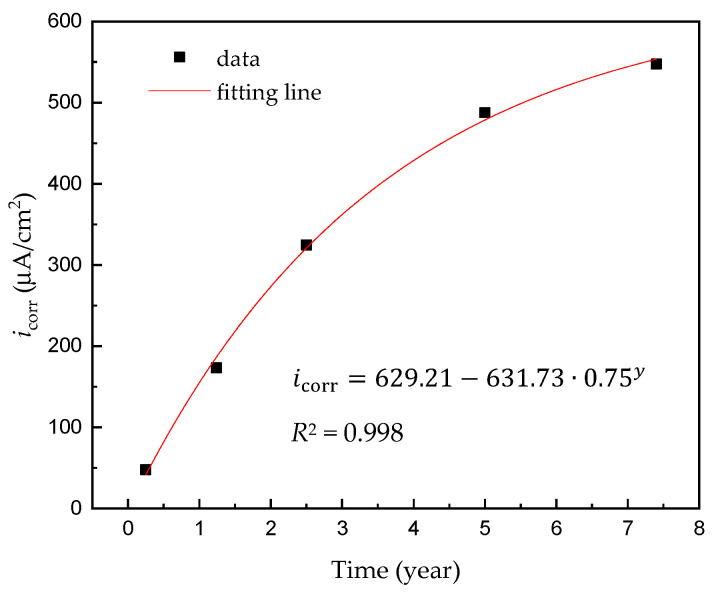
Variation of corrosion current density for carbon steel pipeline in a soil solution according to the acceleration time.

Figure 5 shows the potentiodynamic polarization curves obtained for corroded steel in a synthetic soil solution as a function of the acceleration time. The electrochemical parameters obtained via the polarization test are listed in Table 5. It can be seen that the potentiodynamic polarization curve of the non-rust steel is quite different from those of the corroded steels, implying differences in the reaction process and corrosion mechanism. Normally, when there is no rust layer, the corrosion process of the carbon steel electrode is controlled by oxygen diffusion: if the rust layer hinders the oxygen diffusion process, the corrosion process will be inhibited, results will increase the Tafel slope of the cathodic polarization curve. However, the experimental results show that, when the carbon steel surface is covered with a rust layer, the Tafel slope of the cathodic curve is reduced, indicating that the cathodic process has been promoted. Therefore, it is believed that the rust layer formed by carbon steel in a synthetic soil solution cannot effectively hinder the diffusion of oxygen, thus leading to an acceleration of the corrosion process. As the corrosion time increases, the corrosion current density (*i*_corr_ mA/cm^2^) increases gradually, and *E*_corr_ moves toward positive values. The amount of rust increases even upon increasing the corrosion time; this rust layer not only cannot protect the carbon steel but also accelerates its corrosion further. The corrosion current density values listed in Table 5 are plotted in Figure 6, and a variation in the corrosion current density can be noted according to the corrosion time (*y*/year). In particular, the corrosion current density initially exhibits a continuous increase, but the rate of increase becomes less pronounced after 5 years, i.e., the expected saturation is observed.

**Figure 7:**
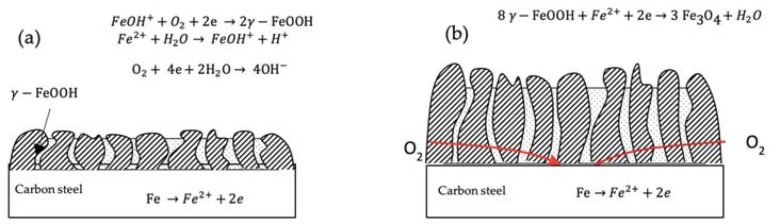
Corrosion mechanism for a buried pipeline according to time: (**a**) initial stage and (**b**) later stage.

The corrosion process shown in Figure 7 explains the corrosion mechanism of pipeline steel in the synthetic soil solution. The rusting of ferrous is an electrochemical process that begins with the transfer of electrons from iron to oxygen. Ferrous is the reducing agent, whereas oxygen is the oxidizing agent. The rate of corrosion is affected by electrolytes. The reaction is the reduction of oxygen [16]:

(2) $$O_{2}+2H_{2}O\;+4e\to 4OH^{-}$$

The electrons for the above reaction are provided by the oxidation of iron, which may be described as follows [16]:

(3) $$\mathrm{Fe}\to {\mathrm{Fe}}^{2+}+2e$$

The ferrous ions (Fe^2+)^ will form hydrated ions in solution [16]:

(4) $${\mathrm{Fe}}^{2+}+H_{2}O\to {\mathrm{FeOH}}^{+}+H^{+}$$

The intermediate corrosion product FeOH^+^ can be oxidized quickly by O_2_ and converted into lepidocrocite (γ−FeOOH), the brown rust γ −FeOOH accumulates on the metal surface and forms the early rust layer (the outer rust layer) according to [17]:

(5) $${\mathrm{FeOH}}^{+}+O_{2}+2e\to 2\gamma -\mathrm{FeOOH}$$

Hence, γ −FeOOH accumulates on the surface of the metal and forms the early rust layer. The γ −FeOOH layer, which is in electrical contact with the metal, can be reduced according to [16,17,18]:

(6) $$8\;\gamma -\mathrm{FeOOH}+{\mathrm{Fe}}^{2+}+2e\to 3{\mathrm{Fe}}_{3}O_{4}+4H_{2}O$$

When the reduction potential of γ −FeOOH is higher than the corrosion potential of carbon steel in the solution, γ −FeOOH can be reduced and works as an oxidant. Fe_3_O_4_ can accumulate at the interface between the γ −FeOOH and the surface metal, forming the inner rust layer. In previous works, it has been reported that ferrous ions and electrons can pass through Fe_3_O_4_, and hence the cathodic reaction can occur on the surface of the Fe_3_O_4_ [16,19]. Additionally, the value of the oxygen diffusion coefficient (*D*) it has been proved that oxygen is reduced on the surface of the rust layer. Therefore, it can be concluded that the Fe_3_O_4_ layer is a large cathode area, and oxygen is reduced on its surface therefore the corrosion rate of carbon steel can be promoted significantly. Other studies have demonstrated that, when the metal corrosion rate is high, this rate can be controlled by limiting the diffusion of the oxidant. This is because when the outer rust layer of γ −FeOOH loosens and drifts out of the soil solution, this layer cannot hinder the diffusion process of oxygen effectively [20]. Corrosion at the surface is determined by the rate of oxygen diffusion into the pores of the Fe_3_O_4_ layer; once equilibrium is reached, the corrosion current density is saturated [18]. The limiting diffusion current density can be described as follows [16,21]:

(7) $$i_{L}=-nFD\frac{C_{0}}{\delta }$$

where *D* is the diffusion coefficient of oxygen, *C*_0_ is the concentration of oxygen in the solution and δ is the thickness of the diffusion layer. All of the parameters in the above equation were kept constant with all experimental condition unchanged, therefore $i_{L}$ has a specific value and the corrosion rate of carbon steel stabilizes at a certain value. It was found that, while such scales exhibit high electrochemical activity, difficulties in using in-situ spectroscopic techniques for electrochemical experiments have led to a lack of agreement in the reaction schemes proposed by different authors. Consequently, the importance of reaction (b.1) during the corrosion process of iron or carbon steel in soil could not be clarified until very recently, and this reaction is extracted from articles on atmospheric corrosion, not exactly in articles on soil corrosion. However, corrosion in soil is aqueous, and the mechanism is electrochemical, but the conditions in the soil can range from ‘atmospheric’ to ‘completely immersed’ [22]. Likewise, reaction (b.1) can explain why the corrosion current density is saturated in this study when corrosion occurs in soil over a long time exposure; the main component of corrosion products steel pipelines in soil are γ −FeOOH and Fe_3_O_4_.

**Figure 8:**
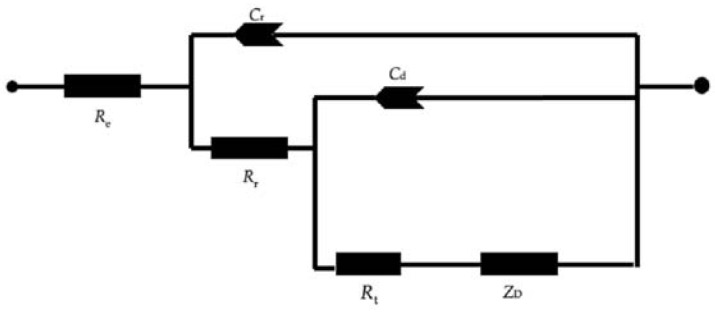
The equivalent electrical circuit for carbon steel/solution products/water interphase is used to describe how rust adheres to metal surfaces. *R*_e_: electrolyte resistance, *C*_f_: film capacity, *R*_f_: electrolyte resistance across the film, *C*_d_: double layer capacity, *R*_t_: charge transfer resistance, *Z*_D_: diffusional impedance.

**Figure 9:**
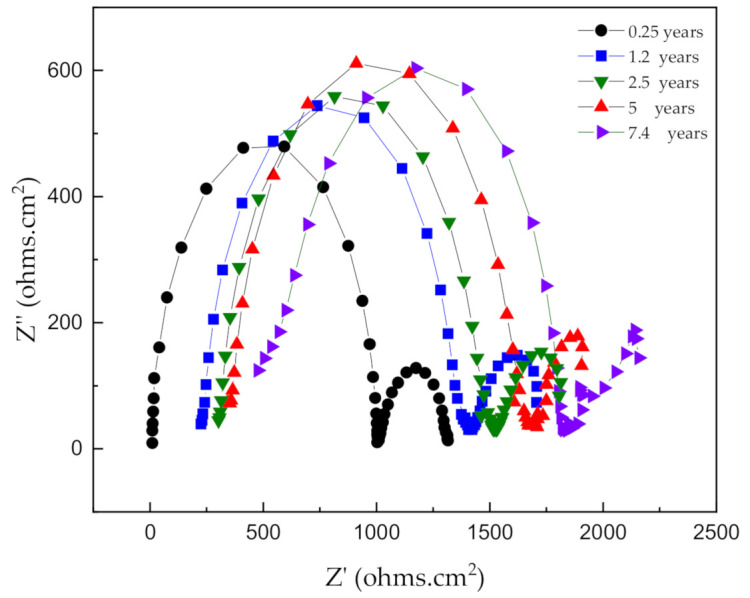
Impedance spectra are presented in the form of a Nyquist plot.

The properties of the rust layer were further investigated via in-situ EIS measurements to obtain real-time corrosion data in a continuous process. Figure 8 illustrates the equivalent circuit used for a short immersion time, which was used to fit the data [19,20]. At very high-frequency (HF), the imaginary component, Z’’, disappears, leaving only the electrolyte resistance *R*_e_. For the 0.25 years case, the outer rust layer of γ −FeOOH remains on the surface of the electrode; this data can be fitted using the circuit in Figure 8. However, the results of the Nyquist plot, as shown in Figure 9, describe an increase of the HF resistance with increasing corrosion time. This is because, after a certain period of corrosion time, the amount of rust created is quite large and the exposure area is small (0.25 cm^2^). Thus, not all the rust (especially γ −FeOOH) can cling to the metal surface; it gradually loosens and drifts out of the solution. Hence, the fitting value of the HF resistance corresponds to not only the *R*_e_ value but it is replaced the sum of *R*_e_ and *R*_0_, the latter being the resistance of the layer which grows with the immersion time (i.e., *R*_HF_ = *R*_e_ + *R*_0_) [21].

**Figure 10:**
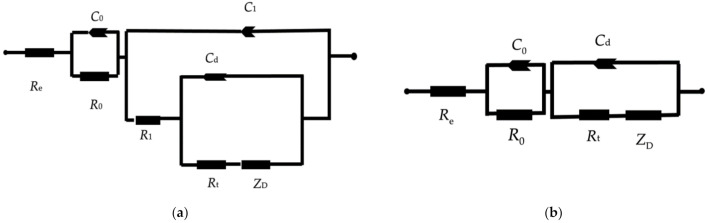
The equivalent electrical circuits for the carbon steel/corrosion products/soil solution interface: (**a**) a portion of the rust loosens out of the solution; (**b**) a portion of the rust (Fe_3_O_4_) adheres to the metal surfaces. *R*_e_: electrolyte resistance, *C*_0,_ and *C*_1_: film capacitance, *R*_0,_ and *R*_1_: film resistance, *C*_d_: double layer capacity, *R*_t_: charge-transfer resistance, *Z*_D_: diffusion impedance.

The equivalent circuit was then modified with *R*_0_ and *C*_0_, where *C*_0_ is the layer capacitance, whose low value is out of the range of the frequency domain used for the measurements. This new equivalent circuit is shown in Figure 10a. This modification of the equivalent circuit means that the interface is coated by two layers characterized by the pairs of parameters *R*_0_, *C*_0,_ and *R*_1_, *C*_1_. The increase of the *R*_0_ value with time corresponds to the increase of the thickness of the porous brown γ −FeOOH external layer. The equivalent circuit in Figure 10a can be fitted for the 1.2, 2.5, and 5 years cases [20]. The *R*_1_ value is almost constant, with a mean value of 1000 Ω·cm^2^. Furthermore, the experiments with 1.2, 2.5, and 5 years of corrosion times at low frequencies are complicated by the influence of the γ −FeOOH rust on the metal surface, with a proportion of it being drifted out of the solution. The equivalent circuit, shown in Figure 10b, is only adequate for the 7.4 years case. After this corrosion time, almost all brown rust scale loosens and leaches out of the solution, and the main component of rust which remains on the metal surface is black rust (Fe_3_O_4_) [16]. Since the Fe_3_O_4_ layer cannot be distinguished from the remaining metal via electrical measurements, owing to its good electronic conductivity [20], it was believed that the thick inner rust Fe_3_O_4_ layer could not be detected via EIS measurements. Consequently, it was inferred that the corrosion rate of carbon steel in a synthetic soil solution was determined by the limiting diffusion rate of oxygen. Hence, the equivalent circuit is shown in Figure 10b is proposed for the rusted electrodes. At low frequency, determining the resistance under the resistors of the surface rust is a very complex process, probably due to the oxygen capability to diffuse into the pores. As long as oxygen is saturated inside the black rust, the corrosion rate of carbon steel remains stable at a certain value. This is still reasonable and uniform in the case of actual underground environments. When the black rust increases rapidly, the scale is too thick, the outer layer will break the structure that clings to the metal surface, it will also gradually loosen and expose the inner rust layer in direct contact with the soil environment.

### 3.4. Required Current

A series of studies have been performed to examine the influence of soil conductivity on cathodic protection systems applied to the protection of buried pipelines [23]. As can be seen in the following two equations, the total current increases due to the increase in soil conductivity:

(8) I=237k

where 2 ≤ *k* ≤ 60 and:

(9) $$I=1895\cdot e^{0.0373\cdot k}$$

where 61 ≤ *k* ≤ 120.

**Figure 11:**
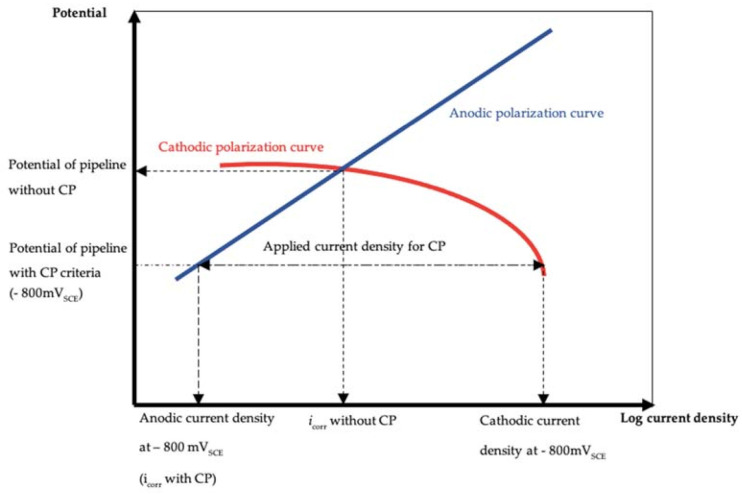
Evan’s diagram, indicating the relationship between the applied current density and protective potential [7,24,25].

Here, *I* is the total required current in mA, and *k* is the soil conductivity in mS/m. These two equations govern the relationship between the total current required for protection and the conductivity and are valid within two separate regimes. Indeed, Equation (8) is a linear expression, whereas Equation (9) is an exponential relationship. However, the above two formulas apply to newly buried pipelines and not to pipelines that have been buried for a long time. Because of the corrosion mechanism for buried pipelines, the required current depends not only on the conductivity of the soil but also on the rust covering the cathode. Therefore, the specific required current to be applied for the CP approach depends on the service time of the buried pipeline. To devise a CP strategy, the applied CP current density (*i_app_*) should be calculated using the Evan’s diagram, as shown in Figure 11 [7,24,25].

**Figure 12:**
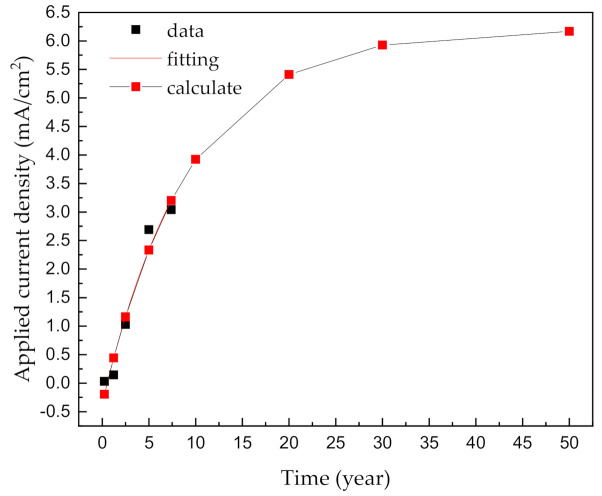
Applied current density as a function of the corrosion time for steel pipelines in a soil solution.

As in previous studies, the applied current density was calculated as the difference between the anodic polarization curve and the cathodic polarization curve at −800 m*V_SCE_* (the protective potential for C-steel) [26]. The corresponding calculation results are shown in Table 6, and the values of the applied current density as a function of the corrosion time are plotted in Figure 12. The obtained *R*^2^ value of 0.96 suggests that the fitting is reliable, and the empirical equation attained after an analysis of the fitting is:

(10) $$i_{app}=6.2-6.6\times 0.9^{y}$$

As can be seen from Figure 12, according to the equation corresponding to a corrosion time exceeding 50 years, *i_app_* approaches the maximum value of 6.2 mA/cm^2^. The purpose of this study is to assess the electrical current applied to an underground pipe. Under standard DNV-RP-B401, the required current, *I_req_*, needs to provide an adequate polarizing capacity and maintain cathodic protection during the design life. *I_req_* can be calculated from the individual surface area, *A*, coating breakdown factor, *f_c_*, and relevant design current density, *i_c_*, if applicable [26]:

(11) $$I_{req}=A\cdot i_{c}\cdot f_{c}$$

The coating breakdown factor describes the anticipated reduction in the cathodic current due to the application of an electrically insulating coating. When *f_c_* = 0, the coating is 100% electrically insulating, and the cathodic current density thus decreases to zero. In contrast, *f_c_* = 1 means that the coating has no current-reducing properties. In general, the relevant design current density can be considered to be the applied current density value that has been measured above. Equation (11) may be written as:

(12) $$I_{req}=A\cdot \left(6.2-6.6\times 0.9^{y}\right)$$

Equation (12) should be applied to design a CP system for buried pipeline steel, according to the pipeline service time and surface area.

## 4. Conclusions

In this study, electrochemical tests were performed on pre-buried pipelines to optimize the required current needed for the CP strategy according to the pipeline service time. This research is of great interest in engineering applications by a novel conducted study. According to the results, the following conclusions can be pointed out:

- The corrosion current density of pipeline steel in a soil environment reaches a stable and saturated state after a long time, due to the oxygen limited diffusion rate in the oxide layer at equilibrium.
- When designing a CP system for buried pipeline steel, the required CP current should be determined according to the logarithmic equation derived in this study.

## Figures and Tables

**Table 1:** Chemical composition of SPW400 (wt.%).

Fe	C	P	S
Bal.	0.25 max	0.04 max.	0.004 max.

**Table 2:** Chemical composition of the synthetic soil solution (ppm).

CaCl_2_ (ppm)	MgSO_4_·7H_2_O (ppm)	NaHCO_3_ (ppm)	H_2_SO_4_ (ppm)	HNO_3_ (ppm)
133.2	59.0	208.0	48.0	21.8

**Table 3:** Potentiodynamic polarization results for pipeline steel.

*E*_corr_ (m*V_SCE_*)	*i*_corr_ (µA/cm^2^)	Corrosion Rate (mm/year)
−676.8	4.2	0.098

**Table 4:** Conditions for accelerated corrosion tests as a function of the service time.

**Un-Accelerated Condition**			**Accelerated Condition**	
Corrosion Current Density (μA/cm^2^)	4.2	→	Accelerating Current Density (μA/cm^2^)	5000
Real time for corrosion	0.25 years	→	Accelerated time for corrosion	1.8 h
	1.2 years	→		9 h
	2.5 years			18 h
	5 years			36 h
	7.4 years	→		54 h

**Table 5:** Potentiodynamic polarization results of pipeline steel as a function of the acceleration time.

Acceleration Time. (year)	Potential (m*V_SCE_*)	*i*_corr_ $\left(\mu A/{\mathrm{cm}}^{2}\right)$	*β_a_* $\left(mV_{\mathrm{SCE}}\right)$	*β_c_* $\left(mV_{\mathrm{SCE}}\right)$
0	−676.8	4.2	127.9	365.5
0.25	−732.4	47.7	282.6	200.2
1.2	−722.5	173.2	386.2	227.1
2.5	−644.6	324.6	317.5	257.7
5	−579.3	487.6	258.4	261.5
7.4	−499.2	547.6	316.9	323.2

**Table 6:** Results for the calculation of parameters: the anodic current density and applied current density are based on the experiment results.

Acceleration Time (year)	Anodic Current Density at −800 m*V_SCE_* $\left(\mu A/{\mathrm{cm}}^{2}\right)$	Cathodic Current Density at −800 m*V_SCE_* $\left(\mu A/{\mathrm{cm}}^{2}\right)$	Applied Current Density $\left(\mathrm{mA}/{\mathrm{cm}}^{2}\right)$
0.25	47.5	79.8	0.0323
1.2	173.0	317.8	0.1448
2.5	324.1	1355.4	1.0313
5	486.7	3177.3	2.6906
7.4	546.7	3589.7	3.0430

## Data Availability

Data sharing is not applicable to this article.
